# A cross-sectional network analysis of courage, fresh start mindset, and depression across gender and educational stage in adolescents

**DOI:** 10.3389/fpsyg.2026.1851629

**Published:** 2026-06-04

**Authors:** Kaiying He, Yaoyao Cai, Danling Zhan, Yongxiang Yang, Jie He, Siyi Chen, Kaixin Chen, Lishen Wang

**Affiliations:** 1 School of Educational Science, Shaoguan University, Shaoguan, Guangdong, China; 2 Mental Health Education and Counseling Center, Shaoguan University, Shaoguan, Guangdong, China

**Keywords:** adolescent depression, bridge centrality, courage, educational stage, fresh start mindset, gender differences, network analysis

## Abstract

**Background:**

Adolescent depression is a major public health concern. While most research has focused on risk factors, less is known about how protective psychological resources work together. This study examined the network of associations between courage, fresh start mindset (FSM), and depression, and whether the network configuration differed across gender and educational stage.

**Methods:**

A total of 18,484 Chinese adolescents completed measures of depression, courage, and FSM. We estimated Gaussian graphical models using graphical LASSO regularization, with model selection guided by the Extended Bayesian Information Criterion. Network Comparison Tests were used to compare network structure and global strength across gender and educational stage. Bridge expected influence was estimated under both theory-driven and data-driven community structures.

**Results:**

Across all networks, depression shared negative associations with FSM and multiple courage dimensions, while the courage dimensions and FSM were positively interconnected. Network structure varied significantly by gender and educational stage, yet global strength remained comparable, pointing to differences in the arrangement of specific edges rather than in overall connectivity. Under theory-driven communities, social-oriented responsibility courage (RES_S) exhibited the highest bridge expected influence, and FSM also showed consistently high bridging values. Under data-driven communities, FSM clustered with social courage in boys and junior high school students, but was grouped with depression and individual courage in girls and senior high school students.

**Conclusion:**

Courage and FSM formed a dense network of protective associations. RES_S had the most prominent bridging connections, while FSM acted as a cognitive bridge whose network placement shifted across gender and educational stage. These patterns suggest that the estimated association structure among protective resources is broadly similar in overall connectivity but differs in configuration across developmental and gendered contexts, providing directions for future longitudinal and intervention research.

## Introduction

Adolescent depression is a major public health concern, with prevalence rates increasing sharply from early adolescence onward (Salk et al., 2017; Shorey et al., 2022; Thapar et al., 2022). In China, a recent meta-analysis of 439 studies reported a pooled prevalence of 26.17% for depressive symptoms among children and adolescents (Zhou et al., 2024), a figure that represents an increase from earlier estimates of 24.3% focusing solely on secondary school populations (Tang et al., 2019). Depression in adolescence predicts poorer academic and relational functioning, later mental health problems, and long-term psychosocial difficulties (Clayborne et al., 2021; Copeland et al., 2021). These risks are especially salient during the transition from junior to senior high school in China, where academic pressure and future-oriented evaluations intensify (Liu and Lu, 2011; Sun et al., 2013).

Existing research has focused mainly on risk factors, with less attention to the interplay among protective resources in adolescents. Not all stressed adolescents develop depression, and resilience frameworks posit that adaptation depends on the presence and coordination of protective processes (Luthar et al., 2000; Masten, 2001; Masten et al., 2023; Southwick et al., 2014). Dual-factor models further suggest that wellbeing and psychopathology are partially independent, warranting the study of strengths in their own right (DiLeo et al., 2022; Greenspoon and Saklofske, 2001; Suldo and Shaffer, 2008). To move beyond isolating single predictors, it is necessary to examine how protective resources such as adaptive beliefs, character strengths, and role-based commitments are configured together (Ungar, 2011).

Network analysis provides a suitable framework for this purpose. Network theory models psychological phenomena as systems of interacting components, where nodes—symptoms, beliefs, or behavioral tendencies—can exhibit mutual associations (Borsboom, 2017; Borsboom and Cramer, 2013; Fried et al., 2017). Recently, this approach has been extended to protective factors, allowing researchers to estimate how strengths interconnect and which nodes link different domains (Borsboom et al., 2021; Fried and Robinaugh, 2020). In this study, we adopt a network perspective to map the organizational structure of multiple protective resources in adolescents.

Among such resources, courage has received growing attention. Courage is the willingness to pursue valued goals despite fear, uncertainty, or risk, and serves as a counterweight to the avoidance and passivity that sustain internalizing problems (Pury and Lopez, 2010; Rate et al., 2007). Empirical work has linked courage to wellbeing, life satisfaction, and flourishing, as well as to lower anxiety and depressive symptoms (Magnano et al., 2021; Martínez-Martí and Ruch, 2017; Pajestka and Poraj-Weder, 2025; Rachman, 2004). These associations are especially relevant to adolescent depression, which is often marked by withdrawal, helplessness, and reduced goal-directed behavior—features that courage, by definition, is oriented toward counteracting (Woodard and Pury, 2007; Lodi et al., 2022).

Within Chinese culture, courage has been conceptualized along two orientations: individual-oriented courage and social-oriented courage (Cheng and Huang, 2016). Each orientation includes three facets: perseverance, breakthrough, and responsibility. Perseverance refers to persisting through difficulty, breakthrough reflects acting despite uncertainty or fear, and responsibility involves owning one’s choices and fulfilling obligations to others. This dual-orientation framework reflects the broader distinction between independent and interdependent self-construals that characterizes East Asian psychological functioning (Markus and Kitayama, 1991), and captures the culturally specific ways in which courageous action is understood and valued in Chinese society. This cultural framework is developmentally relevant because adolescence involves both increasing personal agency and continued embeddedness in family, school, and peer expectations (Eccles et al., 1993; Steinberg and Morris, 2001). During adolescence, Chinese youth must navigate both personal autonomy and relational obligations, making the six facets potentially differentially related to depression (Chen, 2012). Examining them separately can clarify which forms of courage are most strongly associated with lower symptoms.

These courage dimensions may be associated with depression through different pathways. Individual-oriented courage may counter helplessness and disengagement by supporting persistence and action under difficulty (Beck, 2008; Chorpita, 2002). From a behavioral activation perspective, the willingness to approach rather than avoid challenging situations is a core mechanism in alleviating depressive states (Dimidjian et al., 2011), and individual-oriented courage may facilitate precisely this kind of approach behavior. Social-oriented courage, in contrast, may promote belonging, role-based meaning, and commitment to others, all of which may support resilience (Ryan and Deci, 2000). Self-determination theory posits that relatedness and competence are fundamental psychological needs, and social-oriented courage may sustain these needs by enabling adolescents to fulfill valued social roles even under pressure (Ryan and Deci, 2000). At the same time, social responsibility may also involve pressure, guilt, or role strain in demanding school contexts—a possibility consistent with research on the costs of excessive obligation and self-sacrifice in collectivist cultural settings (Bedford and Hwang, 2003). Its role in adolescent depression should therefore be examined empirically rather than assumed to be uniformly beneficial.

Another promising protective factor is the fresh start mindset (FSM), the belief that one can move beyond past failures and begin again psychologically (Price et al., 2018). FSM is grounded in research on the “fresh start effect,” which shows that temporal landmarks can help individuals separate their present identity from past shortcomings (Dai et al., 2014; Wilson and Ross, 2001). FSM is related to, but distinct from, other positive beliefs such as optimism and growth mindset. Optimism concerns general positive expectations for the future (Scheier et al., 1994), and growth mindset concerns beliefs about the malleability of abilities or traits (Dweck, 2006; Yeager and Dweck, 2012). FSM, by contrast, focuses specifically on psychological renewal after setbacks. Because depressed adolescents often feel defined by accumulated failures, FSM may help weaken the perceived finality of past events and restore future-oriented engagement, which aligns with cognitive models of depression (Beck, 2008; Nolen-Hoeksema et al., 2008). Evidence links FSM to greater goal re-engagement and reduced rumination (Price et al., 2018), but its role in adolescent depression remains underexplored. Although FSM has rarely been studied in adolescents, its focus on moving beyond past failure may be particularly relevant during adolescence, a period marked by repeated academic and social evaluation.

Integrating FSM and courage is theoretically meaningful. Resilience models highlight the interplay between cognitive reappraisal and behavioral approach (Folkman, 2008; Kalisch et al., 2015). FSM can be viewed as a cognitive resource that facilitates the perception of new beginnings, while courage—particularly its perseverant and breakthrough aspects—facilitates active coping. It is important to note that these constructs are not mutually exclusive: courage itself involves deliberate appraisal (Rate et al., 2007); however, FSM and courage represent distinct protective components that may be differentially associated with depressive symptoms within a network. Understanding which specific nodes link cognitive, motivational, and symptomatic domains may help inform future research on protective structures.

Building on this reasoning, we formulated a priori predictions about which nodes would be most strongly connected across communities. Social-oriented responsibility courage (RES_S) occupies the intersection of individual agency and interpersonal obligation—a position that, in an interdependent cultural setting, may make it a pivotal link between the social courage community and the individual/symptoms community. In contrast, FSM, by targeting the cognitive aftermath of failure, is conceptually positioned at the boundary between maladaptive appraisals (depression) and renewed action (courage). We therefore hypothesized that, under a theory-driven community partition, RES_S and FSM would show relatively high bridge expected influence (Hypothesis 2).

We further expected that the network configuration might vary across demographic groups. Girls report higher rates of depression than boys from early to mid-adolescence onward, a pattern often linked to interpersonal orientation, sensitivity to relational stress, and gender-role socialization (Hankin and Abramson, 2001; Rose and Rudolph, 2006). Research further suggests that girls tend to employ more ruminative coping strategies and place greater emphasis on interpersonal relationships as sources of self-worth, which may shape the relative importance of different protective resources (Nolen-Hoeksema and Girgus, 1994; Rudolph, 2002). It is therefore possible that girls rely more heavily on relational or responsibility-based protective pathways, whereas boys rely more on action- or breakthrough-related pathways. The educational stage may also matter. Compared with junior high school students, senior high school students face more intense pressure concerning examinations, identity formation, and future planning (Casey et al., 2008; Crone and Dahl, 2012; Steinberg, 2005). For younger adolescents, psychological renewal may be more closely connected with external structure and social support, whereas for older adolescents it may be more intertwined with internal self-evaluation and future-oriented reconstruction. Examining these subgroup differences may help clarify whether protective resources remain stable in overall strength but differ in internal organization. We therefore predicted that network structure and data-driven community configurations would differ across gender and educational stage, even if global connectivity (global strength) remained similar (Hypothesis 3).

In summary, the present study aims to: (1) estimate the overall network of six courage dimensions, FSM, and depression; (2) identify bridge nodes under both theory-driven and data-driven communities; and (3) test network invariance across gender and educational stage. Based on the above rationale, we hypothesized that (*H1*) courage dimensions and FSM would be positively interconnected and negatively associated with depression; (*H2*) RES_S and FSM would show relatively high bridge expected influence under the theory-driven community structure; and (*H3*) network structure and community configuration would differ across gender and educational stage, whereas global strength would remain comparable.

## Materials and methods

### Participants and procedure

This study used a cross-sectional design. Participants were recruited through cluster sampling from junior and senior high schools in one district of Shaoguan City, Guangdong Province, China. Data were collected in October 2025 through the Wenjuanxing online survey platform. The survey was part of a school mental health monitoring program organized by the local educational administration. Schools, homeroom teachers, and parents provided informed consent. Students completed the survey anonymously and voluntarily, and they were informed that they could withdraw at any time without penalty. The study followed the Declaration of Helsinki and was approved by the Ethics Committee of Shaoguan University (Approval No. 2026-YXLLSC-004).

A total of 21,516 students completed the survey. Because all items were mandatory in the online system, there were no missing data. To ensure data quality, responses were excluded if they showed obvious patterned responding, if completion time was under 180 s, if the attention-check item was answered incorrectly, or if respondents failed the validity or impression-management checks embedded in the courage scale. After screening, 18,484 valid cases remained for analysis.

The final sample included 9,652 boys (52.2%) and 8,832 girls (47.8%). There were 13,319 junior high school students (72.1%) and 5,165 senior high school students (27.9%).

### Measures

#### Fresh start mindset

FSM was measured with the six-item scale developed by Price et al. (2018). Items are rated on a 7-point Likert scale from 1 (*strongly disagree*) to 7 (*strongly agree*). Higher scores indicate a stronger belief that one can move beyond past failures and make a new beginning. A sample item is “Anyone can make a new start if they want to.”

Given that the FSM scale has not been validated within the Chinese cultural context, we followed a standard translation and back-translation procedure to adapt it into Chinese (Beaton et al., 2000). We then randomly selected a subsample of 1,838 participants (approximately 10% of the full sample) and split it into two independent subsamples for exploratory and confirmatory factor analyses (*n* = 909 and *n* = 929, respectively). In the exploratory subsample, a principal component analysis (PCA) was conducted to explore the underlying structure of the data. The Kaiser-Meyer-Olkin measure of sampling adequacy (KMO = 0.898) and Bartlett’s test of sphericity (*χ^2^* = 3543.718, *df* = 15, *p* < 0.001) confirmed that the data were suitable for such analysis. Using varimax rotation, the retention criteria—including eigenvalues > 1, inspection of the scree plot, and theoretical interpretability—supported a one-component solution. This factor explained 69.83% of the total variance, and all item loadings ranged from 0.798 to 0.895. The composite reliability was 0.933, and the average variance extracted was 0.698. The CFA further supported the one-factor model: χ^2^ = 19.725, *df* = 7, Comparative Fit Index (*CFI*) = 0.996, Tucker-Lewis Index (*TLI*) = 0.992, Root Mean Square Error of Approximation (RMSEA) = 0.044, 90% CI [0.022, 0.068], Standardized Root Mean Square Residual (*SRMR*) = 0.014. These findings support the structural validity of the Chinese version of the FSM scale in the present sample. In this study, McDonald’s ω for the FSM scale was 0.909. Full-sample CFA and measurement invariance results across gender and educational stage are reported in Supplementary material; these analyses supported the one-factor structure and full scalar invariance of the FSM scale.

#### Courage

Courage was measured with the Chinese Courage Inventory (CCI; Cheng and Huang, 2016). The CCI distinguishes between individual-oriented courage and social-oriented courage. Each domain includes three facets: perseverance courage, breakthrough courage, and responsibility courage. All substantive items are rated on a 5-point Likert scale from 1 (*strongly disagree*) to 5 (*strongly agree*). The full inventory also includes impression-management items and one validity item.

The present study focused on the six dimensions of courage proposed by Cheng and Huang (2016). These dimensions comprise two orientations: individual-oriented courage [perseverance (PER_I), breakthrough (BT_I), and responsibility (RES_I)] and social-oriented courage [perseverance (PER_S), breakthrough (BT_S), and responsibility (RES_S)]. Higher scores indicate higher levels of courage in the corresponding domain. In the present sample, McDonald’s ω for the six dimensions were as follows: PER_I = 0.715, BT_I = 0.747, RES_I = 0.686, PER_S = 0.755, BT_S = 0.731, RES_S = 0.755.

#### Depression

Depression was measured with the depression subscale of the Hospital Anxiety and Depression Scale (Zigmond and Snaith, 1983). Each item is rated on a 4-point scale from 0 to 3, with higher total scores indicating more severe depressive symptoms. The HADS-D was chosen over the Patient Health Questionnaire-9 (PHQ-9) for two reasons: (a) the HADS-D excludes somatic symptoms, offering a relatively specific index of anhedonia and negative affect that reduces overlap with non-depressive experiences common in school settings (e.g., fatigue, sleep disturbance due to academic stress); and (b) the PHQ-9 is routinely administered in Chinese school-based mental health screening, making many students repeatedly familiar with its items, which may increase the risk of patterned or socially desirable responding. The less familiar HADS-D was therefore selected to mitigate these potential response biases. The HADS has been validated in Chinese adolescent samples (Chan et al., 2010). In the present study, McDonald’s ω for the depression subscale was 0.697. Although this value indicates only modest internal consistency, we retained the scale because its coverage of core affective symptoms aligns well with the non-clinical nature of the sample; the implications of this modest reliability for network estimation are addressed in the Discussion.

### Data analysis

Initial data screening and preliminary analyses, including descriptive statistics, mean difference tests (independent *t*-tests and one-way ANOVAs), and Pearson correlations, were conducted in SPSS 26.0.

To evaluate potential common method bias (CMB), we first conducted a confirmatory factor analysis (CFA), conceptually analogous to Harman’s single-factor test, in which all items were loaded onto a single latent factor. This model showed poor fit to the data, χ^2^(1595) = 232,562.18, *p* < 0.001, CFI = 0.518, TLI = 0.500, RMSEA = 0.089, and SRMR = 0.089, indicating that a single factor could not account for the observed covariance structure (Podsakoff et al., 2003). Given the limitations of the single-factor test, we further applied the unmeasured latent method construct (ULMC) approach using a common latent factor (CLF). We first estimated a baseline three-factor CFA model, which yielded a modest fit, χ^2^(776) = 123,738.36, *p* < 0.001, CFI = 0.668, RMSEA = 0.093, SRMR = 0.089. We then added an orthogonal CLF to the baseline model, allowing all items to load on both their substantive constructs and the method factor. The inclusion of the CLF improved model fit, χ^2^(735) = 64,263.60, *p* < 0.001, CFI = 0.829, RMSEA = 0.068, SRMR = 0.044. A Satorra–Bentler scaled chi-square difference test confirmed that this improvement was significant, *Δχ*^2^(41) = 41,466.00, *p* < 0.001. The squared standardized loadings on the CLF indicated that the method factor accounted for an average of 20.39% of the total variance (range = 0.34–60.82%), which is below the 25% benchmark observed by Williams et al. (1989) and the widely recognized 25–50% threshold range for substantive concern (Podsakoff et al., 2003). Thus, while some shared method variance may be present, it is unlikely to fully account for the substantive associations reported in the present study. Nevertheless, given the fully self-report nature of the data, we caution in the Discussion that bridge estimates in particular could still be partially inflated by common method variance.

Network estimation and visualization were conducted in R (version 4.3). We estimated Gaussian graphical models using the *bootnet* and *qgraph* packages (Epskamp et al., 2018). To obtain sparse and interpretable networks, we used graphical LASSO regularization with Extended Bayesian Information Criterion model selection and set the tuning parameter to 0.50. This tuning parameter was selected to balance sparsity and interpretability, consistent with common practice in psychological network analysis. The network included eight nodes: depression (DEP), FSM, and the six courage dimensions. Each node represented a total score for a questionnaire or a subscale. Edges represent regularized partial correlations after controlling for all other nodes. Although network parameters were estimated with regularization to improve interpretability, edge weights and centrality indices should be interpreted as descriptive features of the estimated association network rather than as indicators of causal influence. Networks were visualized with the Fruchterman–Reingold algorithm.

Node importance was indexed by expected influence (EI), which retains the sign of edges and is appropriate for networks that include both positive and negative associations (Robinaugh et al., 2016). To identify nodes linking different domains, we calculated 1-step bridge expected influence using the *networktools* package (Jones et al., 2021).

Bridge analyses were conducted under two types of community structure. In the theory-driven model, the eight nodes were assigned to four communities: depression, FSM, individual courage, and social courage. In the data-driven model, community detection was performed with the Walktrap algorithm based on the absolute edge-weight matrix.

To compare networks across gender and educational stage, we used the Network Comparison Test (van Borkulo et al., 2023) with 1,000 permutations. We tested network structure invariance, global strength invariance, and edge-level differences. For multiple comparisons at the edge and centrality levels, *p*-values were adjusted using the Benjamini–Hochberg false discovery rate procedure.

Network accuracy and stability were assessed using bootstrapped confidence intervals and case-dropping bootstrap procedures. Correlation stability coefficients above 0.25 were considered acceptable, and values above 0.50 were considered good (Epskamp et al., 2018).

## Results

### Descriptive statistics and correlations

Demographic characteristics of the sample are presented in Table 1, and descriptive statistics by gender and educational stage are shown in Tables 2, 3. Boys scored slightly higher than girls on FSM and most courage dimensions, and also reported slightly higher depression scores. Senior high school students reported higher depression scores and lower scores on several individual-oriented courage dimensions than junior high school students. As shown in Table 4, depression was negatively correlated with FSM and all six courage dimensions. FSM was positively correlated with all courage dimensions, with the strongest positive correlations observed among courage facets within the same orientation. Overall, the correlation pattern was broadly consistent with the network estimates reported below.

**TABLE 1:** Demographic characteristics of the participants (*N* = 18,484).

Variables	Category	Frequency (%)
Gender	Male	9,652 (52.2)
	Female	8,832 (47.8)
Educational stage	Junior high school	13,319 (72.1)
	Senior high school	5,165 (27.9)
Boarding status	Boarding student	5,655 (30.6)
	Day student	12,829 (69.4)
Family size	Only child (1 child)	1,655 (9.0)
	2 Children	9,884 (53.5)
	3 or more children	6,945 (37.6)
Father’s education level	Primary school or below	2,699 (14.6)
	Secondary school (junior high, senior high, vocational)	12,034 (65.1)
	Associate degree	3,150 (17.0)
	Bachelor’s degree and above	601 (3.3)
Mother’s education level	Primary school or below	4,311 (23.3)
	Secondary school (junior high, senior high, vocational)	10,840 (58.6)
	Associate degree	2,756 (14.9)
	Bachelor’s degree and above	577 (3.1)

**TABLE 2:** Descriptive statistics for study variables by gender.

Variables	Male (*n* = 9,652)		Female (*n* = 8,832)		Total (*n* = 18,484)	
	*M*	*SD*	*M*	*SD*	*M*	*SD*
Dep	5.12	3.58	4.84	3.59	4.99	3.59
PER_I	19.06	3.82	17.98	3.83	18.55	3.86
BT_I	19.29	3.84	17.99	3.93	18.67	3.94
RES_I	15.89	2.98	15.32	3.09	15.62	3.05
PER_S	19.90	3.70	19.70	3.39	19.81	3.56
BT_S	20.30	3.68	19.50	3.53	19.92	3.63
RES_S	14.82	3.48	14.13	3.21	14.49	3.37
FSM	23.75	4.49	23.50	4.23	23.63	4.37

M, mean; SD, standard deviation; FSM, Fresh Start Mindset; PER_I, individual-oriented perseverance courage; BT_I, individual-oriented breakthrough courage; RES_I, individual-oriented responsibility courage; PER_S, social-oriented perseverance courage; BT_S, social-oriented breakthrough; RES_S, social-oriented responsibility courage; Dep, depression.

**TABLE 3:** Descriptive statistics for study variables by educational stage.

Variables	Junior high school (*n* = 13,319)		Senior high school (*n* = 5,165)		Total (*n* = 18,484)	
	*M*	*SD*	*M*	*SD*	*M*	*SD*
Dep	4.84	3.58	5.36	3.57	4.99	3.59
PER_I	18.73	3.93	18.07	3.64	18.55	3.86
BT_I	18.90	3.98	18.07	3.74	18.67	3.94
RES_I	15.84	3.04	15.05	2.99	15.62	3.05
PER_S	19.71	3.66	20.05	3.25	19.81	3.56
BT_S	19.94	3.71	19.87	3.41	19.92	3.63
RES_S	14.58	3.46	14.25	3.11	14.49	3.37
FSM	23.75	4.44	23.31	4.18	23.63	4.37

**TABLE 4:** Pearson correlations among study variables (*n* = 18,484).

Variables	1	2	3	4	5	6	7	8
1 Dep	1	1	1	1	1	1	1	1
2 PER_I	−0.52***							
3 BT_I	−0.54***	0.80***						
4 RES_I	−0.42***	0.62***	0.68***					
5 PER_S	−0.34***	0.44***	0.43***	0.31***				
6 BT_S	−0.36***	0.52***	0.51***	0.41***	0.56***			
7 RES_S	−0.42***	0.61***	0.63***	0.45***	0.53***	0.70***		
8 FSM	−0.39***	0.47***	0.49***	0.36***	0.37***	0.42***	0.47***	

*** *p* < 0.001.

### Network structure

**FIGURE 1:**
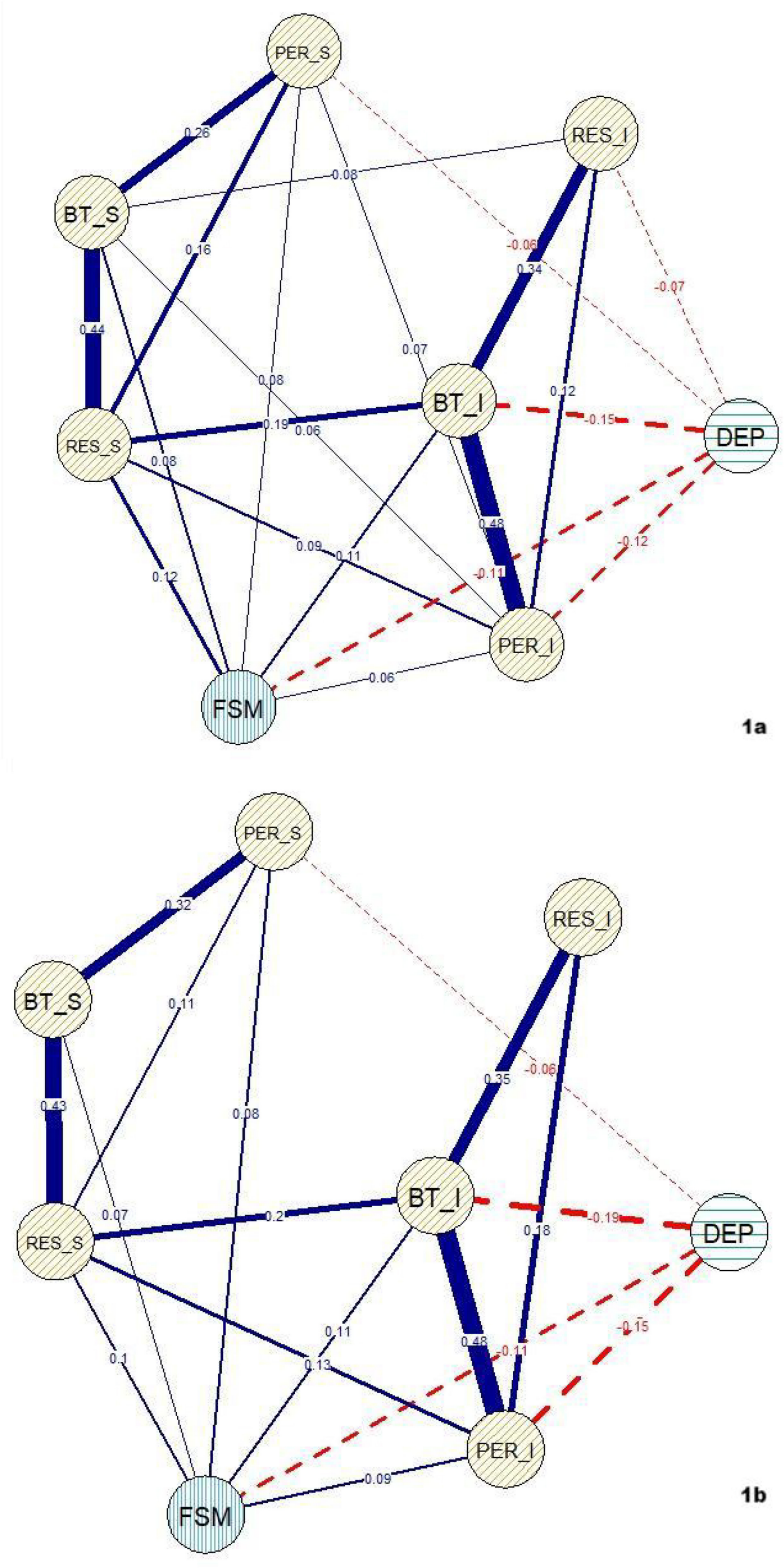
Estimated network structures for male **(a)** and female **(b)** students. Estimated network structures of depression, courage, and FSM in the male and female groups. Nodes represent the study variables (see Table 2 for abbreviations). Edges represent regularized partial correlations after controlling for all other nodes. Blue solid lines indicate positive associations, and red dashed lines indicate negative associations. Thicker and darker edges indicate stronger associations. The networks were estimated using Gaussian graphical models with graphical LASSO regularization and EBIC model selection. Only edges with absolute weights ≥ 0.05 are displayed, and edge labels show the corresponding partial correlation values.

**FIGURE 2:**
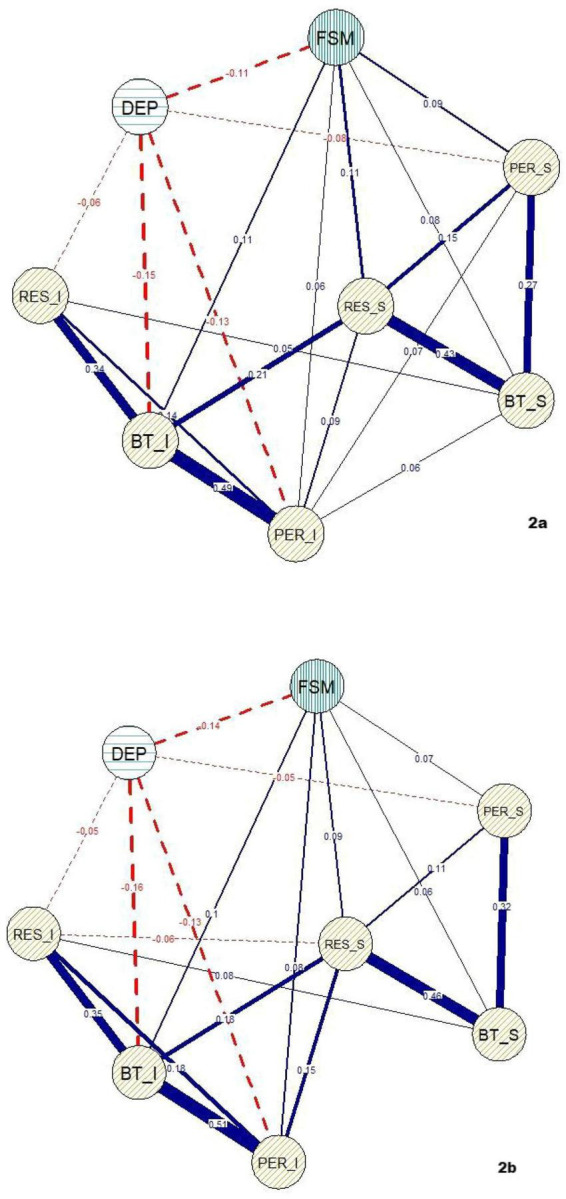
Estimated network structures for junior high school **(a)** and senior high school **(b)** students. Estimated network structures of depression, courage, and FSM in the junior and senior high school groups. Nodes represent the study variables (see Table 2 for abbreviations). Edges represent regularized partial correlations after controlling for all other nodes. Blue solid lines indicate positive associations, and red dashed lines indicate negative associations. Thicker and darker edges indicate stronger associations. The networks were estimated using Gaussian graphical models with graphical LASSO regularization and EBIC model selection. Only edges with absolute weights ≥ 0.05 are displayed, and edge labels show the corresponding partial correlation values.

Estimated Gaussian graphical models for gender and educational stage are presented in Figures 1, 2. Across all four networks, depression showed negative associations with FSM and with multiple courage dimensions. The associations among FSM and courage dimensions were characterized by dense positive connections, especially among dimensions within the same orientation. The subgroup networks were all relatively dense, with 25, 22, 24, and 23 nonzero edges retained in the male, female, junior high school, and senior high school networks, respectively. Visual inspection suggested broad similarity in overall connectivity, but potential differences in the configuration of specific edges required formal network comparison.

### Centrality and stability

**FIGURE 3:**
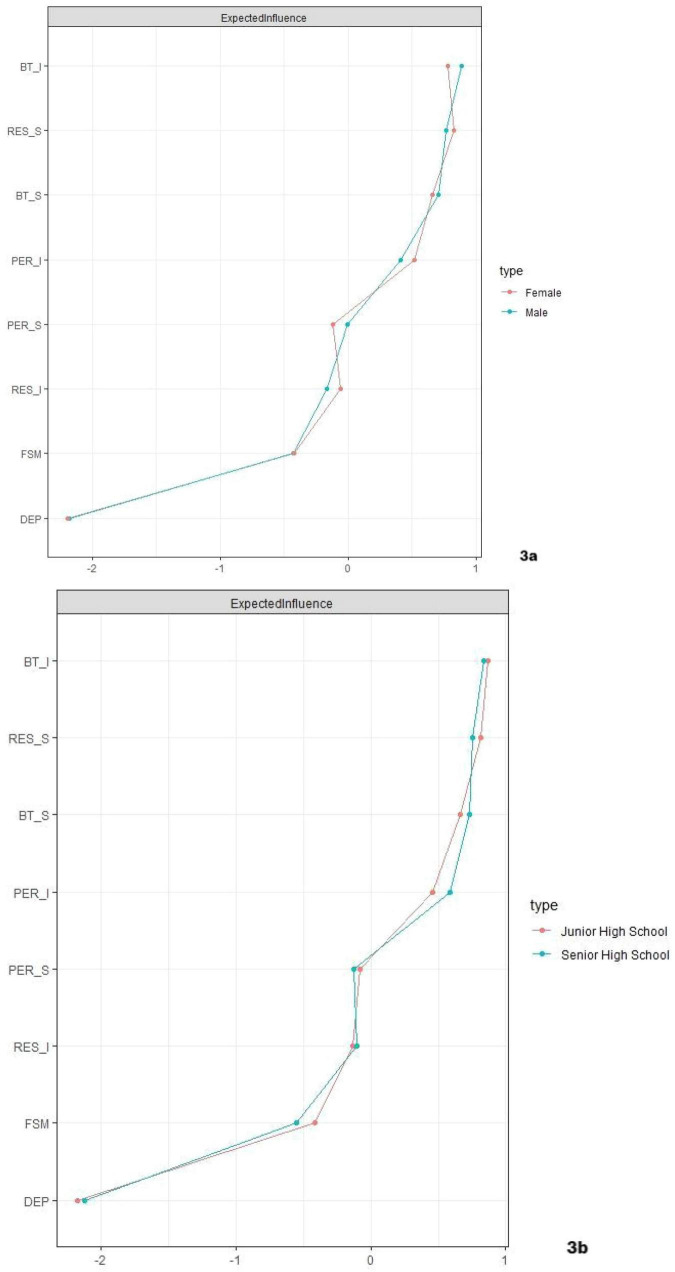
Centrality plots of expected influence by gender and educational stage. **(a)** Comparison between male and female students. **(b)** Comparison between junior and senior high school students. Centrality indices are shown as z-scores.

Expected influence (EI) was used to index node importance (Figure 3). Across groups, BT_I, BT_S, RES_S, and PER_I tended to show relatively higher EI values, whereas FSM occupied a more moderate position in the overall EI ranking. Depression had consistently negative expected influence values in all four networks, indicating that its partial associations with other nodes were predominantly negative. Overall, the EI pattern was highly similar across gender and educational stage.

**FIGURE 4:**
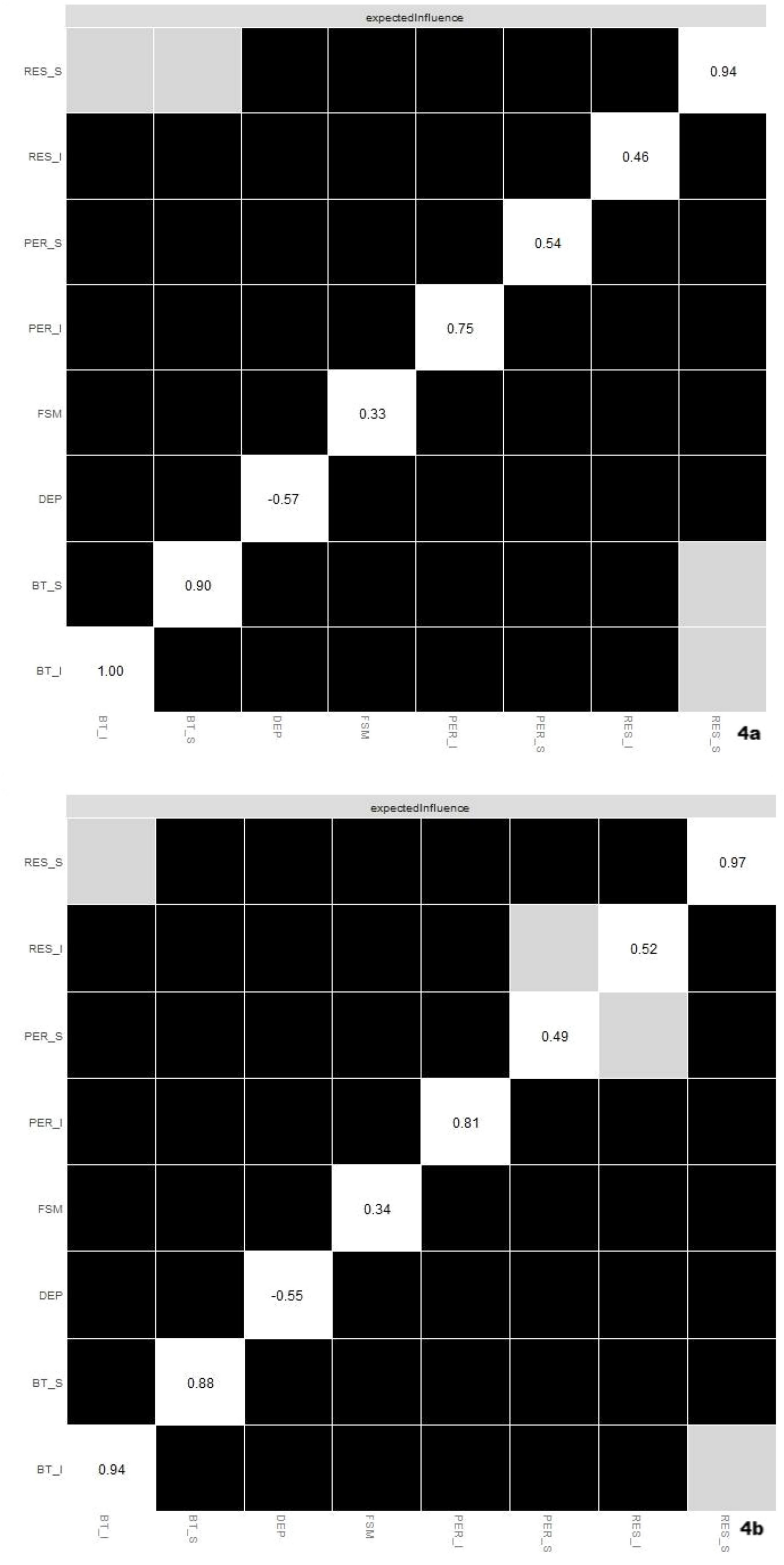
Bootstrapped difference tests for node expected influence in male **(a)** and female **(b)** students networks. Bootstrapped difference tests for node expected influence (EI) in the male and female networks, based on 2,000 nonparametric bootstrap samples. The values in the white boxes on the diagonal represent the original EI values of each node. Off-diagonal boxes represent pairwise comparisons between node EI values. Black boxes indicate statistically significant differences (i.e., the 95% bootstrapped confidence interval of the difference does not include zero), whereas gray boxes indicate non-significant differences.

**FIGURE 5:**
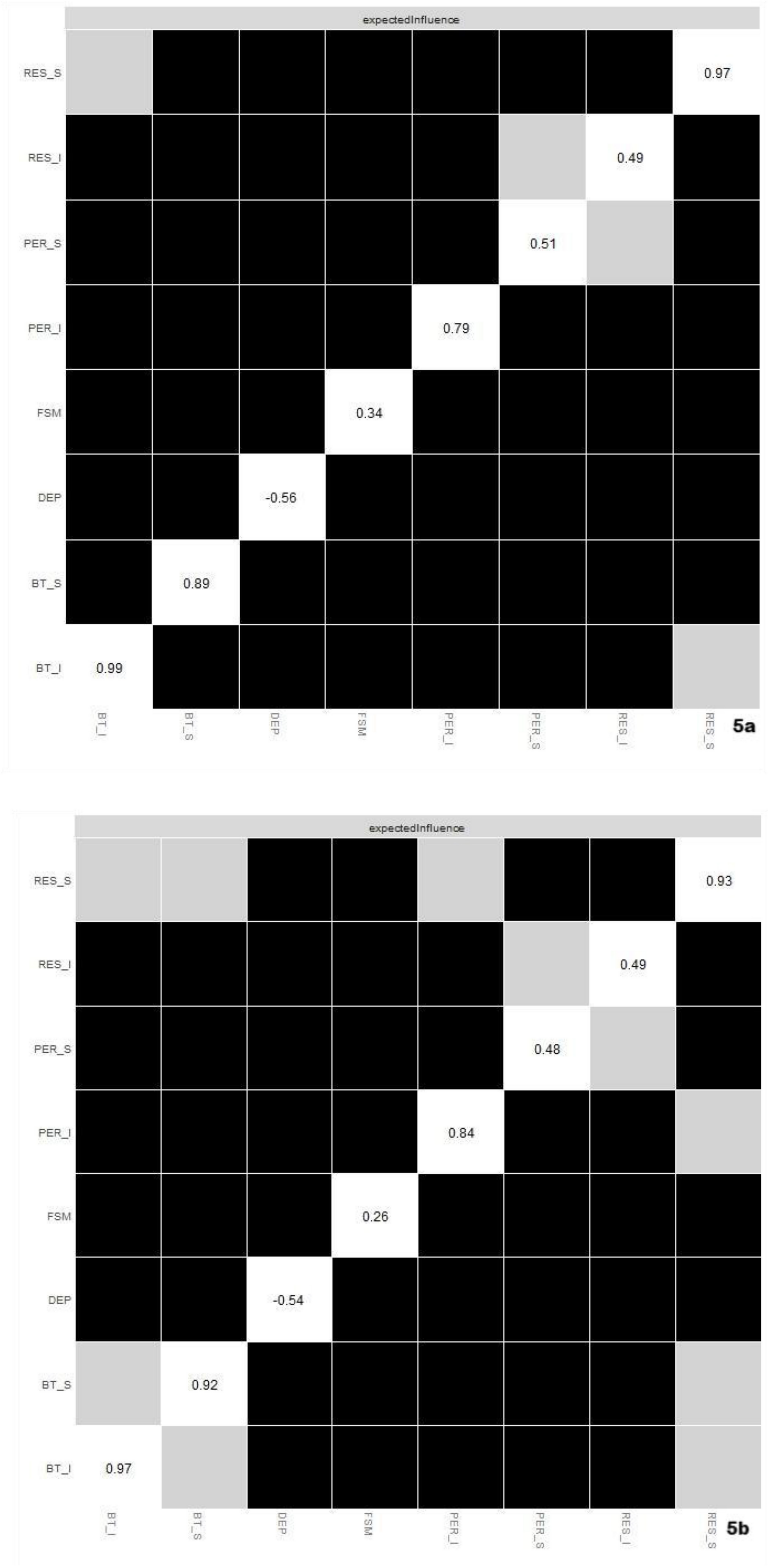
Bootstrapped difference tests for node expected influence in junior high school **(a)** and senior high school **(b)** students networks. Bootstrapped difference tests for node expected influence (EI) in the junior and senior high school networks, based on 2,000 nonparametric bootstrap samples. The values in the white boxes on the diagonal represent the original EI values of each node. Off-diagonal boxes represent pairwise comparisons between node EI values. Black boxes indicate statistically significant differences (i.e., the 95% bootstrapped confidence interval of the difference does not include zero), whereas gray boxes indicate non-significant differences.

**FIGURE 6:**
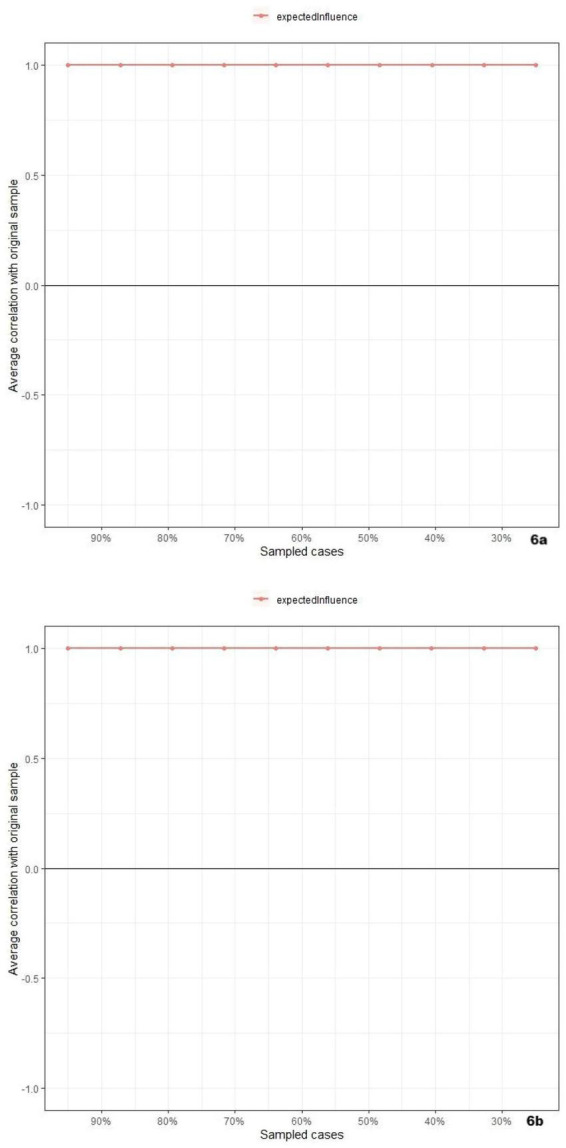
Case-dropping bootstrap stability test for expected influence in male **(a)** and female **(b)** students networks. Case-dropping bootstrap stability test for node expected influence (EI) in the male and female networks. The *x*-axis indicates the proportion of cases dropped from the original sample, and the *y*-axis indicates the correlation between centrality estimates from the subsets and those from the full sample. The red line represents the mean correlation across 2,000 bootstrap iterations, and the shaded area represents the 95% confidence interval. Both networks showed excellent stability, with a CS coefficient of 0.75 for EI.

**FIGURE 7:**
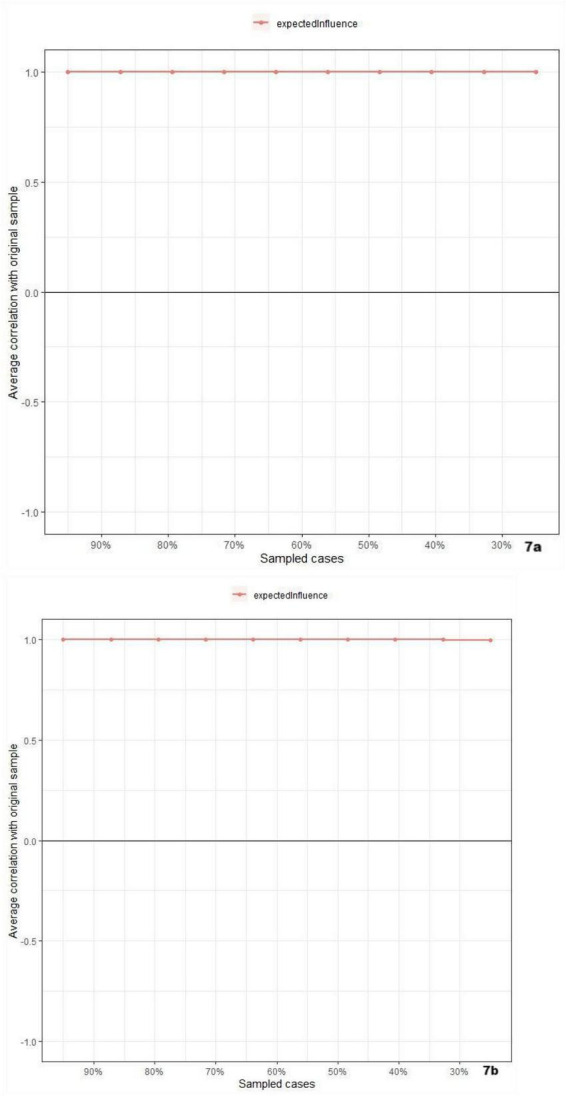
Case-dropping bootstrap stability test for expected influence in junior high school **(a)** and senior high school **(b)** students networks. Case-dropping bootstrap stability test for node expected influence (EI) in the junior and senior high school networks. The *x*-axis indicates the proportion of cases dropped from the original sample, and the *y*-axis indicates the correlation between centrality estimates from the subsets and those from the full sample. The red line represents the mean correlation across 2,000 bootstrap iterations, and the shaded area represents the 95% confidence interval. Both networks showed excellent stability, with a CS coefficient of 0.75 for EI.

Case-dropping bootstrap analyses indicated that EI estimates were stable. The correlation stability (CS) coefficient for EI reached 0.75 in all four networks, exceeding the recommended threshold of 0.50. Bootstrapped difference tests further suggested significant pairwise differences in EI among several nodes (Figures 4–7). These results indicate that the estimated EI patterns were stable within the present sample; however, EI should be interpreted as a relative index of node connectivity rather than as evidence of causal influence.

### Gender differences in network structure

The Network Comparison Test showed that the male and female networks differed significantly in structure but not in global strength (structure invariance: *M* = 0.061, *p* = 0.002; global strength invariance: *S* = 0.039, *p* = 0.380). Thus, gender differences were reflected in the configuration of specific edges rather than in overall connectivity.

After Benjamini-Hochberg correction, eight edges showed significant between-group differences (Table 5). Relative to the male network, the female network showed stronger positive edges between PER_I and RES_I, between PER_S and BT_S, and between PER_I and RES_S, as well as a stronger negative edge between DEP and BT_I. By contrast, the male network showed stronger positive edges between BT_I and PER_S, between RES_I and BT_S, and between PER_S and RES_S. In addition, a direct negative edge between DEP and RES_S was retained only in the male network. These differences suggest that the estimated association structure among courage dimensions and depression varied systematically by gender, even though overall network connectivity was comparable.

**TABLE 5:** Statistically significant edge differences in network comparison tests by gender and educational stage.

Comparison group	Edge (node 1–node 2)	Test statistic (E)	Weight (group 1)	Weight (group 2)
Gender	PER_I–RES_I	0.058	(Male)	(Female)
			0.124	0.182
	BT_I–PER_S	0.027	0.027	0.000
	RES_I–BT_S	0.052	0.077	0.024
	PER_S–BT_S	0.061	0.261	0.321
	DEP–RES_S	0.044	−0.044	0.000
	PER_S–RES_S	0.055	0.160	0.105
	PER_I–RES_S	0.046	0.087	0.133
	DEP–BT_I	0.035	−0.151	−0.186
Educational stage	PER_I–RES_S	0.059	(Junior)	(Senior)
			0.091	0.150
	PER_S–BT_S	0.046	0.272	0.318
	PER_I–PER_S	0.039	0.075	0.035
	PER_I–BT_S	0.055	0.064	0.008
	RES_I–RES_S	0.061	0.000	−0.061

Only edges showing statistically significant between-group differences after Benjamini-Hochberg correction are displayed. E denotes the edge invariance test statistic. Positive values indicate positive partial correlations, and negative values indicate negative partial correlations. For the gender comparison, Group 1 = male and Group 2 = female. For the educational stage comparison, Group 1 = junior high school and Group 2 = senior high school.

### Educational stage differences in network structure

A similar pattern was observed across educational stage. The junior and senior high school networks differed significantly in structure but not in global strength (structure invariance: *M* = 0.061, *p* = 0.009; global strength invariance: *S* = 0.049, *p* = 0.256). Thus, developmental differences were reflected in the arrangement of specific edges rather than in the total level of connectivity.

After Benjamini-Hochberg correction, five edges differed significantly between junior and senior high school students (Table 5). Relative to the junior high school network, the senior high school network showed stronger positive edges between PER_I and RES_S and between PER_S and BT_S. In contrast, the edges linking PER_I with PER_S and BT_S were weaker in the senior high school group. In addition, a negative edge between RES_I and RES_S was retained only in the senior high school network. These findings indicate that the estimated network configuration varied across educational stage, particularly in the way individual and social courage dimensions were interconnected, without a corresponding change in global connectivity.

### Bridge centrality under theory-driven communities

To examine how nodes linked distinct parts of the network, we estimated 1-step bridge expected influence (BEI) under the predefined four-community structure. Across all four networks, RES_S showed the highest positive BEI. FSM also showed consistently high BEI, ranking second in the male, female, and junior high school networks and remaining among the higher bridge nodes in the senior high school network. In contrast, depression showed negative BEI in all four networks. The positive bridging role of RES_S was strongest in the female and junior high school networks (BEI = 0.428 and 0.387, respectively). These results suggest that RES_S and FSM occupied prominent bridging positions in the estimated association network under the theory-driven community specification.

### Data-driven community detection

When communities were identified using the Walktrap algorithm, a more differentiated pattern emerged (Tables 6, 7). In the male and junior high school networks, FSM clustered with the three social courage nodes, whereas depression clustered with the three individual-oriented courage nodes. Under this empirical community structure, BT_I and PER_I showed the strongest positive bridge effects between the two communities (e.g., in the male network, BEI = 0.322 and 0.272, respectively).

**TABLE 6:** Community assignments and bridge expected influence across genders and methods.

Node	Male				Female			
	Theory-driven		Data-driven		Theory-driven		Data-driven	
	C	BEI	C	BEI	C	BEI	C	BEI
DEP	1	−0.566	2	−0.226	1	−0.549	1	−0.063
PER_I	2	0.148	2	0.272	2	0.153	1	0.215
BT_I	2	0.171	2	0.322	2	0.121	1	0.197
RES_I	2	−0.005	2	0.060	2	−0.006	1	0.024
PER_S	3	0.122	1	0.044	3	0.066	2	0.066
BT_S	3	0.199	1	0.119	3	0.130	2	0.130
RES_S	3	0.332	1	0.214	3	0.428	2	0.428
FSM	4	0.327	1	0.050	4	0.340	1	0.251

C, community; BEI, bridge expected influence (1-step). In the theory-driven approach, communities were specified a priori as 1 = symptoms, 2 = individual-oriented courage, 3 = social-oriented courage, and 4 = cognitive characteristics. In the data-driven approach, communities were identified empirically using the Walktrap algorithm. In the male network, 1 = social-oriented courage + FSM and 2 = depression + individual-oriented courage. In the female network, 1 = depression + individual-oriented courage + FSM and 2 = social-oriented courage.

**TABLE 7:** Community assignments and bridge expected influence across educational stage and methods.

Node	Junior high school				Senior high school			
	Theory-driven		Data-driven		Theory-driven		Data-driven	
	C	BEI	C	BEI	C	BEI	C	BEI
DEP	1	−**0.558**	2	−**0.226**	1	−0.537	1	−0.052
PER_I	2	0.162	2	**0.289**	2	0.151	1	0.194
BT_I	2	0.166	2	**0.315**	2	0.114	1	0.181
RES_I	2	0.001	2	0.057	2	−0.035	1	0.020
PER_S	3	0.090	1	−0.003	3	0.054	2	0.054
BT_S	3	0.187	1	0.109	3	0.144	2	0.144
RES_S	3	**0.387**	1	0.273	3	0.364	2	**0.364**
FSM	4	**0.341**	1	0.056	4	0.263	1	**0.220**

C, community; BEI, bridge expected influence (1-step). In the theory-driven approach, communities were specified a priori as 1 = symptoms, 2 = individual-oriented courage, 3 = social-oriented courage, and 4 = cognitive characteristics. In the data-driven approach, communities were identified empirically using the Walktrap algorithm. In the junior high school network, 1 = social-oriented courage + FSM and 2 = depression + individual-oriented courage. In the senior high school network, 1 = depression + individual-oriented courage + FSM and 2 = social-oriented courage. Bold values indicate the highest bridge expected influence within each network.

By contrast, in the female and senior high school networks, FSM clustered with depression and the individual-oriented courage nodes, whereas the three social-oriented courage nodes formed a separate community. Under this structure, RES_S was the strongest positive bridge node (female: BEI = 0.428; senior high school: BEI = 0.364), and the negative bridge effect of depression was substantially attenuated. Overall, the Walktrap analyses revealed a systematic shift in the community location of FSM across gender and educational stage.

Taken together, these results indicate that the estimated position of FSM within the network depended on the community structure derived from the data and varied across groups. In summary, although the theory-driven analysis consistently placed RES_S and FSM in bridging roles, the data-driven analysis revealed that the empirical clustering of FSM differed by gender and educational stage. Group differences were therefore reflected more in network configuration than in overall connectivity.

## Discussion

The present study examined how courage and fresh start mindset (FSM) are associated with adolescent depression within an estimated network framework. Three main findings emerged. First, consistent with Hypothesis 1, courage dimensions and FSM exhibited dense positive interconnections, and depression showed negative associations with FSM and multiple courage dimensions. Second, supporting Hypothesis 2, bridge analyses identified social-oriented responsibility courage (RES_S) as the node with the highest bridge expected influence across all four theory-driven networks, alongside consistently high bridge expected influence for FSM. Third, consistent with Hypothesis 3, network structure differed significantly by gender and educational stage, whereas global strength did not. Data-driven analyses further revealed that the community placement of FSM varied systematically across groups. Collectively, these findings indicate that the organization of these protective resources is relatively stable in overall connectivity but variable in internal configuration. Given the cross-sectional design and exclusive reliance on self-report measures, these patterns are discussed as descriptive features of the estimated association network rather than as causal mechanisms or functional pathways.

A first notable finding was the dense positive connectivity among courage dimensions and FSM. This pattern is consistent with resilience frameworks, which emphasize that adaptation depends on the coordination of multiple protective resources rather than on any single factor (Masten, 2014; Rutter, 2012). It also aligns with the network perspective that protective factors can be modeled as nodes within a psychological network, rather than as background correlates of symptom levels (Borsboom et al., 2021; Fried and Robinaugh, 2020). Furthermore, this finding is congruent with the dual-factor model, which posits that wellbeing and psychopathology are partially independent dimensions (Suldo and Shaffer, 2008). The dense interconnections among protective nodes suggest that these resources may form a relatively distinct set of positive associations that operate alongside—rather than simply as the inverse of—depressive symptomatology. In the present study, FSM was embedded within this broader protective structure, whereas depression was negatively connected to multiple protective nodes across groups. This pattern supports the view that lower depression is associated not only with isolated strengths, but also to the patterning of those strengths into an interconnected network—a conclusion consistent with dynamic systems perspectives on resilience (Kalisch et al., 2015; Ungar, 2011).

Node importance was not evenly distributed across the network. BT_I, BT_S, RES_S, and PER_I exhibited relatively high expected influence across groups, indicating that the estimated network was primarily organized around action-oriented and responsibility-related courage components. The prominence of breakthrough courage is noteworthy in light of the behavioral activation framework discussed earlier: the willingness to approach rather than avoid challenging situations has been identified as a core mechanism for alleviating depressive states (Dimidjian et al., 2011), and the high expected influence of BT_I and BT_S suggests that this approach-oriented function may occupy a central position within the broader protective network. FSM occupied a more moderate position in expected influence, yet this did not diminish its theoretical significance. In the bridge analyses, FSM consistently served as a key cross-domain linking node. This distinction is important because expected influence and bridge expected influence index different network functions (Jones et al., 2021; Robinaugh et al., 2016). Expected influence captures a node’s overall connectivity within the network, whereas bridge expected influence captures its role in linking distinct communities. Accordingly, FSM may be better understood as a cross-community linking node than as a local hub.

The bridge centrality results are central to the interpretation of the study. Across gender and educational stage, RES_S exhibited the highest bridge expected influence in the theory-driven model, and FSM consistently showed high bridge expected influence among the cognitive variables. These findings support Hypothesis 2 and are consistent with the theoretical framework proposed in the Introduction, which suggested that the estimated network could exhibit connections to depression through two complementary sets of associations: one social and role-based, the other cognitive and renewal-based. Crucially, these two bridges occupied distinct yet complementary positions. RES_S was positioned as a node connecting socially embedded forms of courage (PER_S, BT_S) to individual-oriented courage and, through these associations, to depressive symptoms. Its high bridge expected influence indicates that responsibility within meaningful social roles was the dimension most strongly linking the social courage community to other parts of the network. In contrast, FSM was positioned as a cognitive node connecting the belief in the possibility of psychological renewal to the motivational and behavioral components of courage (e.g., perseverance, breakthrough). This complementarity between FSM and courage parallels the distinction between appraisal-focused and action-focused processes highlighted in resilience models (Folkman, 2008; Kalisch et al., 2015; Southwick et al., 2014), while acknowledging that courage itself involves deliberate cognitive appraisal (Rate et al., 2007).

The role of FSM as a bridge node carries theoretical significance. Depression is often maintained by repetitive attention to failure, loss, and negative self-evaluation (Beck, 2008; Nolen-Hoeksema et al., 2008; Watkins, 2008). A fresh start mindset may weaken the perceived finality of past setbacks and restore future-oriented engagement. This interpretation is consistent with Price et al. (2018), who conceptualized FSM as a belief that individuals can move beyond prior failure rather than remain defined by it. Importantly, this function is conceptually distinct from both optimism and growth mindset. Whereas optimism pertains to general positive expectations (Scheier et al., 1994) and growth mindset pertains to beliefs about the malleability of abilities (Dweck, 2006; Yeager and Dweck, 2012), FSM specifically addresses whether the self can be psychologically renewed after setbacks. The present bridge findings suggest that this renewal-specific belief may occupy a unique structural position in the protective network, connecting cognitive reappraisal processes with the motivational resources required for re-engagement. In adolescent contexts characterized by repeated evaluation and social comparison, such a belief may be particularly relevant.

The prominence of RES_S deserves equal emphasis. Social responsibility showed the highest bridge expected influence across all four theory-driven networks. Among the dimensions examined, it was the strongest link between the broader protective network and the depression community. A likely explanation is that responsibility connects the adolescent self to meaningful roles, obligations, and relational commitments, and these connections are inversely associated with withdrawal and passivity. This interpretation is compatible with self-determination theory and related developmental perspectives that emphasize the motivational value of meaningful social engagement (Ryan and Deci, 2000). In the Chinese cultural context, where obligation and interpersonal duty are especially salient, the bridging prominence of RES_S is plausible (Markus and Kitayama, 1991; Triandis, 2001). As noted in the Introduction, the dual-orientation framework of Chinese courage reflects the broader distinction between independent and interdependent self-construals (Cheng and Huang, 2016; Markus and Kitayama, 1991), and the bridge prominence of RES_S suggests that the interdependent, socially embedded dimension of courage showed particularly strong connections to other parts of the network in this cultural context. It should be noted, however, that RES_S may capture not only courage per se but also broader relational or belongingness processes, such as the fulfillment of relatedness needs (Ryan and Deci, 2000), which were not independently assessed in this study. Future research that includes separate measures of social connectedness or belongingness could help disentangle whether the bridge role of RES_S reflects a unique “courage” effect or a more general interpersonal protective factor. At the same time, high bridge expected influence does not imply that responsibility is uniformly beneficial; it only indicates that this node occupied a prominent structural position in the estimated network. Under conditions of strong academic pressure, responsibility may also involve burden or conflict—a possibility consistent with research on the costs of excessive obligation in collectivist educational settings (Bedford and Hwang, 2003).

The network comparison results also merit attention. In line with Hypothesis 3, for both gender and educational stage, network structure differed significantly, whereas global strength did not. This pattern directly supports Hypothesis 3 and carries important conceptual implications. It suggests that group differences manifest in the configuration of specific associations rather than in the total amount of connectivity (van Borkulo et al., 2023). In other words, boys and girls, as well as junior and senior high school students, differed not in the overall strength of their protective system connections but in how those systems were organized. This distinction shifts interpretation away from a simple deficit model toward a configurational account, wherein the same set of protective resources may be interconnected through different configurations depending on developmental and gendered contexts.

The gender-specific edge differences are consistent with this configurational view. The female network showed stronger coupling among several protective pathways, including PER_I–RES_I, PER_S–BT_S, and PER_I–RES_S, as well as a stronger negative association between depression and individual breakthrough. The male network showed stronger BT_I–PER_S, RES_I–BT_S, and PER_S–RES_S edges, and only the male network retained a direct negative edge between depression and social responsibility. These findings suggest that depressive functioning in boys may be more directly tied to the weakening of social-role engagement, whereas girls may show tighter coupling between depression and internally organized protective processes. Such an interpretation is broadly consistent with work showing that girls’ distress is often more closely linked to self-evaluation and internal emotional processing (Hankin and Abramson, 2001; Rose and Rudolph, 2006). It also aligns with the gender socialization literature discussed in the Introduction, which suggests that girls tend to employ more ruminative coping strategies and place greater emphasis on interpersonal relationships as sources of self-worth (Nolen-Hoeksema and Girgus, 1994; Rudolph, 2002). The stronger DEP–BT_I edge in the female network may reflect this pattern: for girls, the capacity for individual breakthrough—acting despite fear and uncertainty—may be more tightly coupled with depressive functioning precisely because their self-evaluative orientation makes avoidance more consequential. For boys, the direct DEP–RES_S edge suggests that the erosion of social responsibility may be a more proximal pathway to depressive symptoms, consistent with evidence that boys’ adjustment is more closely tied to agentic and role-based functioning (Rose and Rudolph, 2006).

A similar pattern emerged across educational stages. Junior and senior high school students differed in structure but not in global strength. Compared with the junior high school network, the senior high school network showed stronger PER_I–RES_S and PER_S–BT_S edges, weaker PER_I–PER_S and PER_I–BT_S edges, and a negative RES_I–RES_S edge that was absent in junior high school students. These findings suggest developmental reorganization rather than simple gain or loss of protection. As adolescents move into senior high school, the relations among personal action, social obligation, and emotional functioning may become more differentiated. The emergence of a negative RES_I–RES_S edge in the senior high school network is particularly noteworthy. It suggests that, for older adolescents, individual and social forms of responsibility may come into tension rather than mutual reinforcement—a pattern that may reflect the intensifying conflict between personal autonomy and social obligation during later adolescence (Chen, 2012; Steinberg and Morris, 2001). In the Chinese educational context, where senior high school students face high-stakes examinations and increasingly consequential future planning (Sun et al., 2013), fulfilling social responsibilities may sometimes compete with individually oriented goals, creating a structural tension that is absent in younger students. This interpretation fits developmental accounts which describe adolescence as a period of increasing reorganization in cognitive, motivational, and social systems (Casey et al., 2008; Crone and Dahl, 2012; Steinberg and Morris, 2001).

The data-driven community results constitute the most distinctive contribution of the present study. In boys and junior high school students, FSM clustered with the three social courage nodes, whereas depression clustered with the individual courage nodes. In girls and senior high school students, FSM clustered with depression and individual courage, whereas social courage formed a separate community. This recurring pattern suggests that the role of FSM is not fixed, but depends on the system in which it is embedded. In boys and younger adolescents, fresh-start beliefs may be supported more strongly by social routines, external structure, or relationally organized forms of adjustment. In girls and older adolescents, however, the clustering of FSM with depression and individual-oriented courage points toward a process of internalization that aligns with key developmental and gender-specific challenges. During later adolescence, particularly for girls, identity exploration intensifies, along with heightened self-focus, rumination, and sensitivity to self-evaluation (Hankin and Abramson, 2001; Nolen-Hoeksema et al., 2008; Steinberg, 2005). Within this context, the belief in a “fresh start” may become more tightly integrated with internal self-regulatory processes (i.e., individual-oriented courage as the capacity to persist and act despite difficulty) and, when these resources are insufficient, with depressive functioning. For these adolescents, FSM may thus function as a psychologically meaningful renewal-related belief that is more internally embedded in the broader network of self-evaluation and coping.

This interpretation is consistent with the conceptual distinction drawn in the Introduction between FSM and related constructs such as growth mindset and optimism (Dweck, 2006; Price et al., 2018; Scheier et al., 1994). Whereas growth mindset addresses whether abilities can change, FSM addresses whether the self can be psychologically renewed. The data-driven community findings suggest that this renewal function may be differentially embedded across groups: externally scaffolded through social processes in boys and younger adolescents, but internally integrated with self-evaluative and coping processes in girls and older adolescents. One plausible interpretation is that, across development and in contexts marked by stronger self-focused processing, fresh-start beliefs become more internalized. This developmental shift may also reflect the increasing capacity for cognitive reappraisal that accompanies prefrontal maturation during adolescence (Casey et al., 2008; Crone and Dahl, 2012), enabling older adolescents to deploy FSM as an internal strategy rather than relying on external temporal landmarks. More broadly, this finding supports the network view that the function of a psychological resource depends partly on its pattern of relations with other nodes (Borsboom, 2017; Borsboom and Cramer, 2013).

The findings also have implications for future intervention research. Because RES_S and FSM showed the most prominent bridge properties in the estimated network, they may be useful candidates for investigation in longitudinal and intervention designs. However, the data-driven community results suggest that these candidates may need to be studied differently depending on developmental stage and gender. For boys and junior high school students, where FSM clusters with social courage, future research might examine whether interventions that activate fresh-start beliefs through structured group activities, collective rituals (e.g., school-wide campaigns marking new semesters), or role-based responsibilities are associated with network reorganization. For girls and senior high school students, where FSM clusters with depression and individual courage, a different approach may be warranted. Here, future research might explore whether cognitive restructuring that positions FSM as an internal resource for managing self-evaluation relates to changes in estimated network structure. More broadly, the present findings suggest that longitudinal research on adolescent depression may benefit from a network-informed perspective—one that tracks not only changes in symptoms directly, but also changes in the interconnections among protective resources. Nonetheless, these suggestions should be regarded as hypotheses for future investigation rather than as evidence of intervention targets, given the cross-sectional design.

Several limitations should be acknowledged. First, the cross-sectional design precludes causal inference. Consequently, all descriptions of “bridge nodes,” “structural positions,” or “network configurations” in this paper refer to patterns of statistical associations in the estimated networks, not to causal mechanisms or functional pathways. These patterns can generate hypotheses about how protective resources may be organized, but they do not demonstrate that any node causally influences another or that targeting a specific node would produce network-wide changes. Longitudinal and experimental designs are needed to test such hypotheses. Second, common method bias remains a concern despite the ULMC analysis suggesting that shared method variance is unlikely to fully account for the results; bridge estimates in particular may still be partially inflated. Third, the modest internal consistency of the HADS-D (ω = 0.697) may have attenuated edges involving the depression node, meaning that true associations could be stronger than those estimated here. Fourth, the study was conducted in a Chinese cultural context; cross-cultural comparisons with Western courage frameworks (e.g., the bravery facet of the VIA Inventory of Strengths) would help determine whether the prominent bridge role of social-oriented responsibility is culturally universal or specific to collectivist settings. In addition, the regularized network approach used here should be interpreted with caution. Because LASSO promotes sparsity, some weak but potentially meaningful edges may have been excluded; thus, the observed density should be understood as an estimate under regularization rather than the full underlying association structure.

In sum, the present study indicates that courage and FSM form an interconnected network of protective associations with adolescent depression. RES_S emerged as the strongest bridge node, while FSM emerged as a stable cognitive bridge whose network position shifted across gender and educational stage. These findings extend the resilience literature by demonstrating that protective resources are not merely additive predictors of lower depression, but form an organized pattern of associations with identifiable bridge nodes that vary in configuration across developmental and gendered contexts. Given the cross-sectional design, these findings are best regarded as descriptive network patterns that can inform hypothesis generation for future longitudinal and experimental investigations.

## Conclusion

In conclusion, the present study demonstrates that courage and fresh start mindset (FSM) are linked in a network of protective associations with adolescent depression. Across gender and educational stage, the overall connectivity of this network remained broadly similar, whereas its internal organization differed significantly. This pattern suggests that the estimated network is comparable in overall strength but distinct in configuration across subgroups.

Two findings deserve particular emphasis. First, under the theory-driven community model, social-oriented responsibility courage (RES_S) showed the highest bridge expected influence across all four networks, and FSM showed consistently high bridge expected influence. This indicates that social responsibility was the node most strongly connecting different communities in the estimated network, whereas FSM was positioned as a cognitive node linking renewal-related beliefs with other protective processes. Second, under the data-driven community model, the community placement of FSM varied systematically across groups: it clustered with social courage in boys and junior high school students, but with depression and individual courage in girls and senior high school students. This finding suggests that the network context of FSM differs meaningfully across developmental and gendered settings.

Taken together, these findings extend network-based research on protective factors by showing that the structure of protective associations can involve both stable bridging patterns and group-specific configurations. From an applied perspective, future longitudinal and intervention research may benefit from examining nodes with high bridge centrality—particularly social responsibility and fresh-start beliefs—to better understand how changes in one part of the network may be associated with changes in other parts over time.

## Data Availability

The raw data supporting the conclusions of this article are available from the corresponding author upon reasonable request.
