# Supplementary material for: A cross-sectional network analysis of courage, fresh start mindset, and depression across gender and educational stage in adolescents
Source: Front Psychol. 2026 Jun 4;17:1851629. doi: 10.3389/fpsyg.2026.1851629 (PMC13275402; doi:10.3389/fpsyg.2026.1851629)
Supplement: Supplementary file 1 [file Table_1.docx]

Supplementary Material

**Full-Sample Confirmatory Factor Analysis and Measurement Invariance of the Fresh Start Mindset Scale**

**Confirmatory Factor Analysis on the Full Sample**

A confirmatory factor analysis (CFA) was conducted on the full analytic sample (N = 18,484) to evaluate the one-factor structure of the Fresh Start Mindset (FSM) scale. The model contained six indicators loading on a single latent factor. The fit indices showed a pattern characteristic of a well-fitting model constrained by small degrees of freedom (*df* = 9) and a very large sample size. The standardized root mean square residual (SRMR = .029) and comparative fit index (CFI = .964) indicated excellent fit, with the Tucker–Lewis index (TLI = .941) and goodness-of-fit index (GFI = .957) providing further support. The root mean square error of approximation (RMSEA = .121, 90% CI [.117, .125]) was elevated; however, this is a known statistical artifact when RMSEA is estimated in models with small degrees of freedom and very large samples (Kenny, Kaniskan, & McCoach, 2015). Following the two-index strategy recommended by Hu and Bentler (1999), the combination of SRMR < .08 and CFI > .95 provides strong evidence for the acceptability of the one-factor model. Table S1 presents the full set of fit indices.

**Table S1** Full-Sample Confirmatory Factor Analysis Fit Indices for the Fresh Start Mindset Scale (N = 18,484)

| Fit Index | Value |
| --- | --- |
| χ² | 2455.804 |
| *df* | 9 |
| *p* | < .001 |
| CFI | .964 |
| TLI | .941 |
| SRMR | .029 |
| RMSEA [90% CI] | .121 [.117, .125] |
| GFI | .957 |

*Note.* CFI = comparative fit index; TLI = Tucker–Lewis index; SRMR = standardized root mean square residual; RMSEA = root mean square error of approximation; GFI = goodness-of-fit index.

**Measurement Invariance Across Gender and Educational Stage**

Multi-group CFA was performed to test measurement invariance of the FSM scale across gender (male vs. female) and educational stage (junior high vs. senior high). Three nested models were examined sequentially: configural invariance (identical factor structure across groups), metric invariance (equal factor loadings), and scalar invariance (equal item intercepts). Given the documented inflation of RMSEA in models with small degrees of freedom and large samples, model comparisons relied on changes in CFI (ΔCFI). Following Cheung and Rensvold (2002) and Chen (2007), a decrease in CFI smaller than .01 was interpreted as evidence that the more constrained model did not meaningfully worsen fit. Table S2 displays fit indices for all models.

**Table S2** Measurement Invariance Fit Indices for the Fresh Start Mindset Scale Across Gender and Educational Stage

| Model | χ²(df) | CFI | TLI | SRMR | RMSEA | ΔCFI |
| --- | --- | --- | --- | --- | --- | --- |
| Gender |  |  |  |  |  |  |
| M1: Configural | 2518.974(18) | .964 | .940 | .029 | .123 | — |
| M2: Metric | 2546.838(23) | .964 | .952 | .031 | .109 | .000 |
| M3: Scalar | 2837.685(28) | .959 | .957 | .032 | .104 | -.005 |
| Educational stage |  |  |  |  |  |  |
| M1: Configural | 2566.291(18) | .963 | .939 | .029 | .124 | — |
| M2: Metric | 2596.857(23) | .963 | .951 | .031 | .110 | .000 |
| M3: Scalar | 2710.592(28) | .961 | .958 | .029 | .102 | -.002 |

*Note.* ΔCFI = change in CFI relative to the preceding, less constrained model. M1 = configural invariance; M2 = metric invariance; M3 = scalar invariance.

**Results**

**Gender.** The configural invariance model (M1) showed good fit, *χ*²(18) = 2518.974, CFI = .964, TLI = .940, SRMR = .029, RMSEA = .123. Constraining factor loadings to equality across males and females (M2) resulted in no decrement in fit, *χ²*(23) = 2546.838, CFI = .964, TLI = .952, SRMR = .031, RMSEA = .109, ΔCFI = .000, supporting metric invariance. The scalar invariance model (M3), which further constrained item intercepts, continued to show strong fit, *χ²*(28) = 2837.685, CFI = .959, TLI = .957, SRMR = .032, RMSEA = .104, ΔCFI = -.005, supporting full scalar invariance.

**Educational stage.** A parallel pattern emerged. The configural model fit well, *χ²*(18) = 2566.291, CFI = .963, TLI = .939, SRMR = .029, RMSEA = .124. Metric invariance held without any loss in CFI, *χ²*(23) = 2596.857, CFI = .963, TLI = .951, SRMR = .031, RMSEA = .110, ΔCFI = .000. Scalar invariance was also supported, *χ²*(28) = 2710.592, CFI = .961, TLI = .958, SRMR = .029, RMSEA = .102, ΔCFI = -.002.

In sum, the FSM scale demonstrated full scalar invariance across both gender and educational stage. These findings justify the use of observed FSM total scores in the between-group network analyses reported in the main manuscript.

Chen, F. F. (2007). Structural Equation Modeling, 14(3), 464–504.

Cheung, G. W., & Rensvold, R. B. (2002). Structural Equation Modeling, 9(2), 233–255.

Kenny, D. A., Kaniskan, B., & McCoach, D. B. (2015). Sociological Methods & Research, 44(3), 486–507.

**Table S2** Results of Independent Samples t-Tests (Gender )

| Variable | ***t*** | ***df*** | ***p*** | **Cohen’s *d*** |
| --- | --- | --- | --- | --- |
| Dep | 5.141 | 18482 | <.001 | 0.076 |
| PER_I | 19.241 | 18482 | <.001 | 0.283 |
| BT_I | 22.633 | 18482 | <.001 | 0.333 |
| RES_I | 12.757 | 18482 | <.001 | 0.188 |
| PER_S | 3.734 | 18482 | <.001 | 0.055 |
| BT_S | 14.979 | 18482 | <.001 | 0.221 |
| RES_S | 13.888 | 18482 | <.001 | 0.205 |
| FSM | 3.830 | 18482 | <.001 | 0.056 |

**Table S3** Results of Independent Samples t-Tests (Educational Stage )

| Variable | *t* | *df* | *p* | *Cohen’s d* |
| --- | --- | --- | --- | --- |
| Dep | -8.865 | 18482 | <.001 | 0.145 |
| PER_I | 10.413 | 18482 | <.001 | 0.171 |
| BT_I | 12.978 | 18482 | <.001 | 0.213 |
| RES_I | 15.857 | 18482 | <.001 | 0.26 |
| PER_S | -5.810 | 18482 | <.001 | 0.095 |
| BT_S | 1.131 | 18482 | \| 0.258 \| \| --- \| | 0.019 |
| RES_S | 5.952 | 18482 | <.001 | 0.098 |
| FSM | 6.226 | 18482 | <.001 | 0.102 |

**Table S4**  Intercorrelations and Effect Sizes (Fisher’s z) Among Study Variables

| **Variable** | **1** | **2** | **3** | **4** | **5** | **6** | **7** | **8** |
| --- | --- | --- | --- | --- | --- | --- | --- | --- |
| 1 Dep | — |  |  |  |  |  |  |  |
| 2 PER_I | −.521 (−0.577) | — |  |  |  |  |  |  |
| 3 BT_I | −.539 (−0.603) | .798 (1.092) | — |  |  |  |  |  |
| 4 RES_I | −.423 (−0.451) | .619 (0.724) | .681 (0.830) | — |  |  |  |  |
| 5 PER_S | −.336 (−0.350) | .438 (0.469) | .425 (0.453) | .314 (0.325) | — |  |  |  |
| 6 BT_S | −.355 (−0.371) | .516 (0.570) | .513 (0.566) | .412 (0.438) | .564 (0.638) | — |  |  |
| 7 RES_S | −.416 (−0.442) | .607 (0.704) | .634 (0.748) | .448 (0.482) | .528 (0.587) | .695 (0.858) | — |  |
| 8 FSM | −.389 (−0.411) | .469 (0.509) | .490 (0.536) | .362 (0.379) | .373 (0.391) | .422 (0.451) | .472 (0.513) | — |

*Note. N* = 18,482. Cells display Pearson correlation coefficient (*r*) with *Fisher’s z* transformation in parentheses. All correlation coefficients were statistically significant at *p* < .001.
